# Antioxidant and Antibacterial Effects of Potential Probiotics Isolated from Korean Fermented Foods

**DOI:** 10.3390/ijms231710062

**Published:** 2022-09-02

**Authors:** Anbazhagan Sathiyaseelan, Kandasamy Saravanakumar, Kiseok Han, Kumar Vishven Naveen, Myeong-Hyeon Wang

**Affiliations:** Department of Bio-Health Convergence, Kangwon National University, Chuncheon 200-701, Korea

**Keywords:** Korean fermented food, probiotics, gut-tolerance, antibiotic susceptibility, hemolysis, antioxidant, antibacterial

## Abstract

A total of sixteen bacterial strains were isolated and identified from the fourteen types of Korean fermented foods that were evaluated for their in vitro probiotic potentials. The results showed the highest survivability for *Bacillus* sp. compared to *Lactobacillus* sp. in simulated gastric pH, and it was found to be maximum for *B. inaquosorum* KNUAS016 (8.25 ± 0.08 log10 CFU/mL) and minimum for *L. sakei* KNUAS019 (0.8 ± 0.02 log10 CFU/mL) at 3 h of incubation. Furthermore, *B. inaquosorum* KNUAS016 and *L. brevis* KNUAS017 also had the highest survival rates of 6.86 ± 0.02 and 5.37 ± 0.01 log10 CFU/mL, respectively, in a simulated intestinal fluid condition at 4 h of incubation. The percentage of autoaggregation at 6 h for *L. sakei* KNUAS019 (66.55 ± 0.33%), *B. tequilensis* KNUAS015 (64.56 ± 0.14%), and *B. inaquosorum* KNUAS016 (61.63 ± 0.19%) was >60%, whereas it was lower for *L. brevis* KNUAS017 (29.98 ± 0.09%). Additionally, *B. subtilis* KNUAS003 showed higher coaggregation at 63.84 ± 0.19% while *B. proteolyticus* KNUAS001 found at 30.02 ± 0.33%. Among them, *Lactobacillus* sp. showed the best non-hemolytic activity. The highest DPPH and ABTS radical scavenging activity was observed in *L. sakei* KNUAS019 (58.25% and 71.88%). The cell-free supernatant of *Lactobacillus* sp. considerably inhibited pathogenic growth, while the cell-free supernatant of *Bacillus* sp. was moderately inhibited when incubated for 24 h. However, the overall results found that *B. subtilis* KNUAS003, *B. proteolyticus* KNUAS012, *L. brevis* KNUAS017, *L. graminis* KNUAS018, and *L. sakei* KNUAS019 were recognized as potential probiotics through different functional and toxicity assessments.

## 1. Introduction

Host–microbiome interactions and their responses regulate homeostasis and diseases through maintaining nutrition, metabolism, immune responses, and circadian rhythmicity [1,2,3,4]. The imbalance of gut microbiomes (beneficial and pathogenic) alter the immune interactions, which cause autoimmune diseases, including gastrointestinal disorders (inflammatory bowel disease and celiac diseases), rheumatic arthritis, malignancy, neurodegenerative disorders, and metabolic syndrome [5,6,7,8,9,10]. Food habits, overuse of antibiotics, and unique genetic characteristics of hosts could alter the gut microbiome [11,12]. Studies have suggested that probiotic supplementation can improve host gut health and prevent auto-immune-related inflammatory diseases [1,13,14]. According to the Food and Agriculture Organization (FAO) and World Health Organization (WHO), adequately ingested probiotics can improve the host’s health [15,16,17].

In the human body (skin, oral, respiratory tract, gut, vagina, and placenta), innumerable microbial species reside in a complex microbial ecosystem (symbiotic to pathogenic) [18,19]. Among them, *Bacteroidetes* (Gram negative) and *Firmicutes* (Gram positive) are found to be higher, while other bacteria (*Actinobacteria*, *Verrucomicrobia* phyla, and *Fusobacteria*) exist on a subdominant level in the adult human gut, but their existence differs in everyone [18,19,20]. Probiotics are frequently isolated from conventional sources (human breast milk, human feces, dairy products) and unconventional sources (non-intestinal, non-dairy fermented food products, different parts of the digestive tracts of various animals) [21,22]. According to FAO and WHO, probiotics should be characterized completely in terms of identification (genus and species level), pathogenicity, antibiotic resistance, resistance to biological barriers (intestinal mucosa and intestinal environment, mucosal surface), and probiotic potential (immunological stimulation, antimicrobial, and antioxidant in vitro and/or in vivo) [23,24]. The gut microbiome develops antibiotic-resistant genes by gut resistome due to frequent exposure to antibiotics [25].

Probiotics isolated from non-intestinal sources, including fermented food, fruit juices, and kimchi, exhibit promising biological properties [21]. Among them, kimchi is a traditional Korean food made by the fermentation process using different leafy and other vegetables (cabbage, radish, cucumber, spinach, green onion, and mustard leaf) alone or together and seafood (anchovy fish and shrimp), seasoned salt, sugar, red chili powder, garlic, and ginger [26]. A study reported that among the numerous microorganisms, lactic acid-producing bacteria (LAB) are richly involved in kimchi preparation. Moreover, the probiotic content and shelf-life of kimchi differ based on the ingredient, storage time, and temperature [27,28]. LAB generally produces beneficial metabolites, such as acids (lactic acid, acetic acid, formic, short-chain fatty acids, etc.), alcohol, aldehydes, ketones, heterocyclic organic compounds (lactocillin, bacteriocins, etc.), enzymes (glycoside hydrolases, proteases), and extracellular polysaccharides [29]. Some isolates of *Leuconostoc* and *Weissella* strains from kimchi are shown to produce biogenic amine and are resistant to streptomycin but are susceptible to other antibiotics [30]; exopolysaccharide from *W. cibaria* exhibited cellular antioxidant activity [31]. Besides, *L. brevis* B13-2 isolate from Korean kimchi was found to have potent probiotic potential and antioxidant activity. In addition, heat-killed bacterial cells had significant antioxidant and immune-modulating ability in RAW 264.7 murine macrophages through the induced expression of IL-1 β, IL-6, TNF-α, and iNOS [32]. Similar to LAB, *Bacillus* species have also been considered probiotics due to their beneficial effects on functional foods, therapeutic potential, and harsh environment tolerance [33,34,35,36]. On the other hand, some *Bacillus* species (*B. anthracis*, *B. thuringiensis*, and *B. cereus*) cause illness to human beings and are considered pathogens [37,38]. However, probiotic/pathogen characteristics of strains are completely strain-specific [39,40]. Furthermore, the industrial and biomedical properties of probiotics vary based on the isolates. Hence, this study aimed to isolate novel bacterial strains with probiotic potential from various Korean fermented fruit juices and Korean kimchi.

## 2. Results and Discussion

### 2.1. Identification of Probiotic Bacterial Strains

About 14 fermented Korean food samples were collected for the isolation of probiotics. Among the samples, except the fermented fruit juices (quince, crimson glory vine, David’s peach, and Japanese apricot green (old)), all other samples were observed the bacterial colonies. Sixteen bacterial isolates were isolated and identified using morphological properties and 16S rRNA gene sequencing primers (27F and 1492R) (Table 1). The results revealed that the 16 isolates were belonging to the genera of *Lactobacillus* (3 strains), *Bacillus* (12 strains), and *Enterobacter* (1 strain). The *Bacillus* sp. includes *B. proteolyticus* KNUAS001, *B. fungorum* KNUAS002, *B. subtilis* KNUAS003, *B. pseudomycoides* KNUAS004, *B. thuringiensis* KNUAS005, *B. bingmayongensis* KNUAS006, *B. luti* KNUAS007, *B. proteolyticus* KNUAS012, *B. wiedmannii* KNUAS013, *B. mojavensis* KNUAS014, *B. tequilensis* KNUAS015, and *B. inaquosorum* KNUAS016. The report supported that *B. subtilis*, *B. coagulans*, and *B. clausii* were commonly used as probiotic strains in many countries [40]. In addition, *B. mojavensis*, reported as an endophytic bacterium, was isolated from the plant *Bacopa monnieri* (Linn.) [41]. *B. inaquosorum* was reported as a sub-species of *B. subtilis* [42]. In this study, *Lactobacillus* sp. was isolated, including *L. brevis* KNUAS017, *L. graminis* KNUAS018, and *L. sakei* KNUAS019. Similarly, *L. brevis* B13-2 and *L. sakei* were reported from Chinese cabbage kimchi and young radish kimchi, respectively [32,43]. *Enterobacter* sp. (*E. hormaechei* KNUAS008) was identified in this study. *Enterobacter* sp. is generally recognized as a foodborne pathogen that is also reported in fermented food products [44].

**Figure 1:**
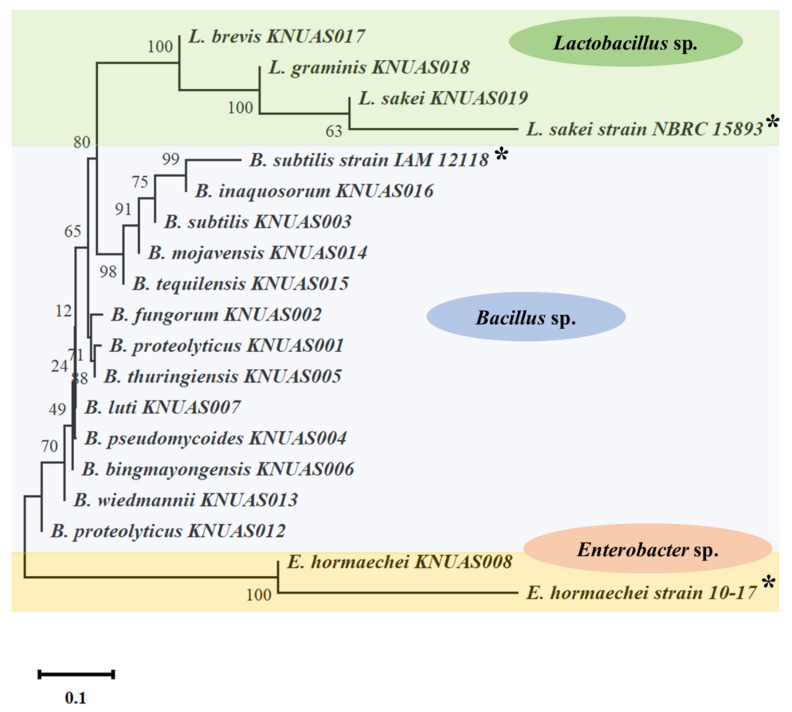
Phylogenetic relationship of 16S rRNA gene sequences of *Lactobacillus*, *Bacillus*, and *Enterobacter* strains were isolated from different Korean fermented foods. Phylogenetic tree constructed by neighbor-joining method. * Reference strains.

Further, the phylogenetic tree displayed a total of three clades belonging to *Bacillus* sp., *Lactobacillus* sp., and *Enterobacter* sp., which indicated that there were close species relationships among the species and respective genera (Figure 1). The 16s rRNA sequencing results indicated that 15 isolates were Gram-positive, while one strain was Gram-negative, which was confirmed with Gram staining analysis. Further, the bromocresol purple assay evidenced that all isolates from *Lactobacillus* sp., produced lactic acid in the MRS agar medium.

### 2.2. Resistance to Biological Barriers

#### 2.2.1. Tolerance in Gastric and Intestinal Fluids

The pH of the human stomach and intestinal fluids ranges from 1 to 4.5 and varies depending upon food consumption, contents, age, and disease condition [45,46]. In addition, the bile salt concentration is a major factor in determining microbial colonization. Survival in gastrointestinal conditions, including low pH and bile salts, is considered an important property of good probiotics. To evaluate the gastrointestinal pH tolerance, bacterial strains were incubated with simulated gastric juice and intestinal fluid at pH 3.0 (Table 2 and Figure S2). The results showed that all the strains survived at the simulated gastric pH for 0–3 h. The survivability ranged from 2.48–7.86 (log10 CFU/mL) and 0.8–8.54 (log10 CFU/mL) at 1 h and 3 h, respectively. Among the strains, the *Bacillus* sp. showed the highest survivability in gastric pH, while cell counts were reduced in *Lactobacillus* sp. (Table 2). In particular, the maximum survival rate of 8.25 ± 0.08 log10 CFU/mL was observed for *B. inaquosorum* KNUAS016 and the minimum survival rate of 0.8 ± 0.02 log10 CFU/mL for *L. sakei* KNUAS019 at 3 h of incubation. For the intestinal fluid tolerance test, the bacterial strains were incubated with bile salts (1%) and pancreatin (0.1%) for 0–4 h at pH 8.0 (Table 2 and Figure S3). The results showed that all the bacterial strains survived for 4 h in intestinal fluid. However, the *Bacillus* sp. exhibited higher survivability compared to *Lactobacillus* sp. (Table 2). Among them, *B. inaquosorum* KNUAS016 and *L. brevis* KNUAS017 had the highest survival rates of 6.86 ± 0.02 and 5.37 ± 0.01 log10 CFU/mL, respectively, in the intestinal fluid environment (Figure S3). According to GRAS status, *B. inaquosorum* was a subspecies of *B. subtilis* and a potential probiotic strain [47]. The earlier report also supported that *L. brevis* KU15153 isolated from kimchi exhibited higher viability under the gastric condition with probiotic potential [48]. Overall, the simulated tolerance studies found that harsh environmental viability highly depends on each strain.

#### 2.2.2. Autoaggregation, Coaggregation, and Hydrophobicity Properties of Bacterial Strains

The autoaggregation and coaggregation ability assays were used to evaluate the potential of probiotics in intestinal colonization through cell-to-cell interaction, biofilm formation, and pathogen inhibition [49]. Hence, all the bacterial strains were tested for their autoaggregation ability at different time intervals (1, 3, and 6 h) (Table 3). All the strains exhibited increased autoaggregation (%) with increasing incubation time from 1 h to 6 h. The maximum autoaggregation (%) was observed at 6 h for *L. sakei* KNUAS019 (66.55 ± 0.33%), *B. tequilensis* KNUAS015 (64.56 ± 0.14%), and *B. inaquosorum* KNUAS016 (61.63 ± 0.19%) but was lower in *L. brevis* KNUAS017 (29.98 ± 0.09%). These results indicated that those bacterial strains could survive and adhere to the gastrointestinal tract. Moreover, the coaggregation ability of bacterial strains was tested at different time intervals (1, 3, and 6 h) (Table 3). At 6 h of incubation, the coaggregation was greater than 50% for *B. subtilis* KNUAS003 (63.84 ± 0.19%), *B. tequilensis* KNUAS015 (63.64 ± 0.35%), *B. mojavensis* KNUAS014 (61.67 ± 0.39%), and *L. sakei* KNUAS019 (55.69 ± 0.30%) but lower for *B. proteolyticus* KNUAS001 (30.02 ± 0.33%). The coaggregation results indicated that *B. subtilis* KNUAS003, *B. tequilensis* KNUAS015, *B. mojavensis* KNUAS014, and *L. sakei* KNUAS019 might have surface proteins that inhibited the pathogens, thereby maintaining the microbial balance in the gut [50].

Further cell surface properties of bacterial strains were determined through hydrophobicity. The hydrophobicity of bacterial strains indicated that they could interact with the intestinal mucosa and epithelial cells, thereby ensuring colonization [51]. Hence, the bacterial strains were incubated with an organic solvent (xylene), and their ability to adhere to the cell surface was tested (Table 3). Interestingly, all *Lactobacillus* spp., including *L. brevis* KNUAS017 (25.64 ± 0.28%), *L. graminis* KNUAS018 (18.81 ± 1.36%)*,* and *L. sakei* KNUAS019 (14.91 ± 0.48%), and one *Bacillus* strain, such as *B. inaquosorum* KNUAS016 (16.76 ± 0.16%), exhibited promising hydrophobicity, while the lowest hydrophobicity was found for *B. bingmayongensis* KNUAS006 (0.09 ± 0.83%) and *E. hormaechei* KNUAS008 (0.24 ± 0.10%). Accordingly, the hydrophobicity may differ with each isolate even within species and with the types of hydrocarbons used in this assay [52].

### 2.3. Safety Assessment

#### 2.3.1. Hemolytic Property

**Figure 2:**
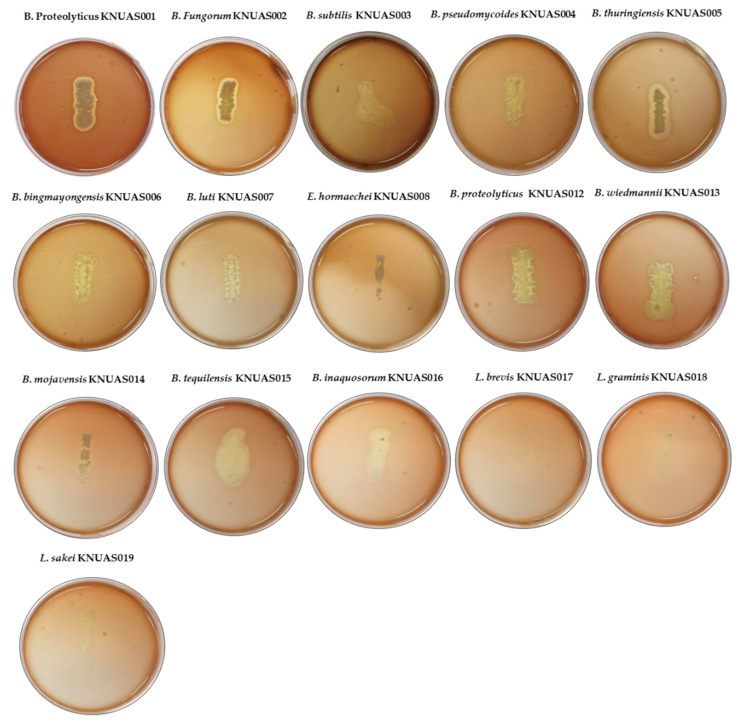
Hemolytic properties of isolated bacterial strains. α-hemolysis, green color in the medium; β-hemolysis, blood lysis, clear zone and γ-hemolysis, no changes in the medium, non-hemolytic.

For the safety assessment, all the bacterial strains isolated from Korean fermented foods were evaluated their hemolytic activity in 5% of sheep blood supplemented blood base agar (Figure 2). Results demonstrated that none of the bacterial strains exhibited the α-hemolysis, while some of the *Bacillus* strains (*B. proteolyticus* KNUAS001, *B. fungorum* KNUAS002, *B. thuringiensis* KNUAS005, and *B. wiedmannii* KNUAS013) showed β-hemolysis. However, the *B. proteolyticus* KNUAS012 did not show significant hemolytic activity compared to *B. proteolyticus* KNUAS001. Accordingly, the study reported that *B. proteolyticus* from Tibetan yaks did not show hemolytic activity but did find probiotic potential [53]. Interestingly, none of the *Lactobacillus* spp. and *Enterobacter* spp. showed any changes, which was considered to be non-hemolytic activity (γ-hemolysis).

#### 2.3.2. Antibiotic Susceptibility

Another safety assessment of probiotics regards antibiotic resistance. According to the FAO and WHO, probiotics should not carry the antibiotic-resistant gene. The results showed that most of the *Bacillus* strains were susceptible to tested antibiotics, but *B. mojavensis* KNUAS014 and *B. inaquosorum* KNUAS016 showed complete resistance to all the tested antibiotics (Table 4 and Figure S4), followed by *B. proteolyticus* KNUAS012, which showed resistance toward vancomycin. The *E. hormaechei* KNUAS008 was susceptible to all the tested antibiotics, but *Enterobacter* sp. caused the pathogenicity and antimicrobial resistance [54]. In addition, the study reported that *E. hormaechei* subsp. Were susceptible to different antibiotics [55]. Interestingly, *L. brevis* KNUAS017 showed resistance to all the tested antibiotics, while *L. graminis* KNUAS018 and *L. sakei* KNUAS019 were susceptible to TCH, ERY, and AMP but resistant to VAN and GEN (Table 4). According to the earlier report, *Lactobacillus* spp. were generally resistant to vancomycin and gentamycin [56,57,58]. Furthermore, antibiotic-resistant genes were found in commercially available health-promoting probiotics [59]. The frequent use of antibiotic-resistant genes containing probiotics may negatively modulate the immune system and impact human health [25]. Most of the LAB strains showed intrinsic resistance to antibiotics that could not be transferred to other microbes. However, the selection of potential health-promoting probiotics is a challenging task that could be overcome by multiple analyses related to the toxicity assessment.

### 2.4. Characterization of Probiotic Potential

#### 2.4.1. Antioxidant Activity

**Figure 3:**
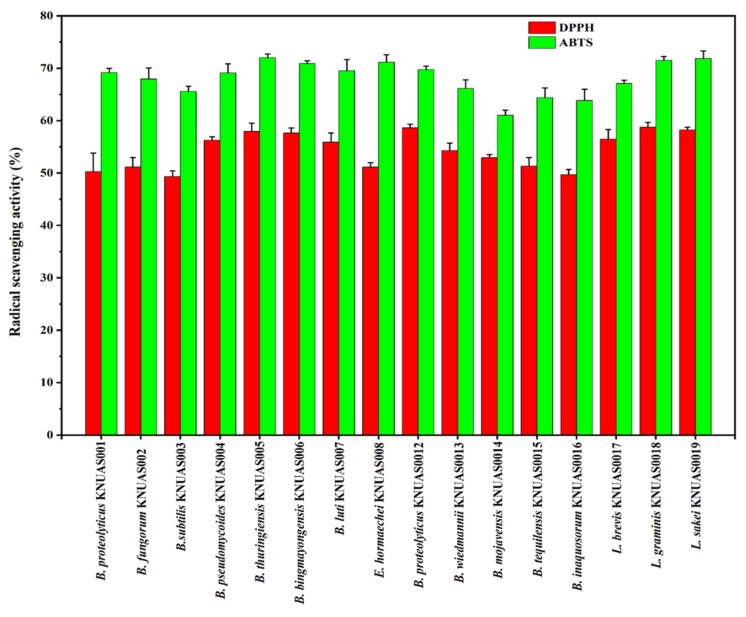
Antioxidant activity of cell free supernatant (CFS) of isolated bacterial strains. DPPH and ABTS radical scavenging activity.

Antioxidant molecules play a major role in retaining the gut microbiome balance by modulating oxidative stress [60]. Evaluating the antioxidant properties of isolated bacterial strains, suspensions were determined in DPPH and ABTS free radical scavenging assay (Figure 3). The results indicated that the bacterial suspensions of all the strains showed a substantial DPPH and ABTS free radical scavenging ability (Figure 3). Among them, the highest DPPH and ABTS radical scavenging activity was observed in *L. sakei* KNUAS019 (58.25% and 71.88%), *L. graminis* KNUAS018 (58.74% and 71.51%), and *B. proteolyticus* KNUAS012 (58.62% and 71.17%). The *B. mojavensis* KNUAS014 showed the lowest ABTS radical scavenging activity at 61.02%. Besides, *B*. *subtilis* KNUAS003 showed the lowest DPPH radical scavenging activity at 49.34%. A study reports that *Lactobacillus plantarum* strains showed <10% DPPH radical scavenging activity [61]. In addition, a previous study reported that *L. plantarum* NJAU-01 significantly decreased lipid peroxidation by increasing enzyme activity in mice [62]. However, the antioxidant activity of isolated *Bacillus* sp. and *Lactobacillus* sp. was considerably good in this study compared to earlier reports [32,63].

#### 2.4.2. Antibacterial Properties

**Figure 4:**
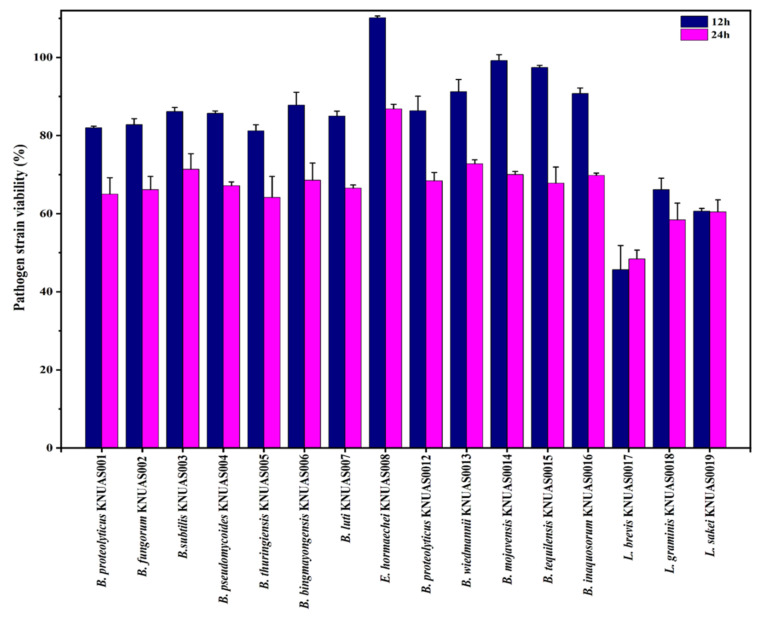
Antibacterial activity of cell-free supernatant of isolated bacterial strains against *Staphylococus aureus* (*S. aureus*).

**Figure 5:**
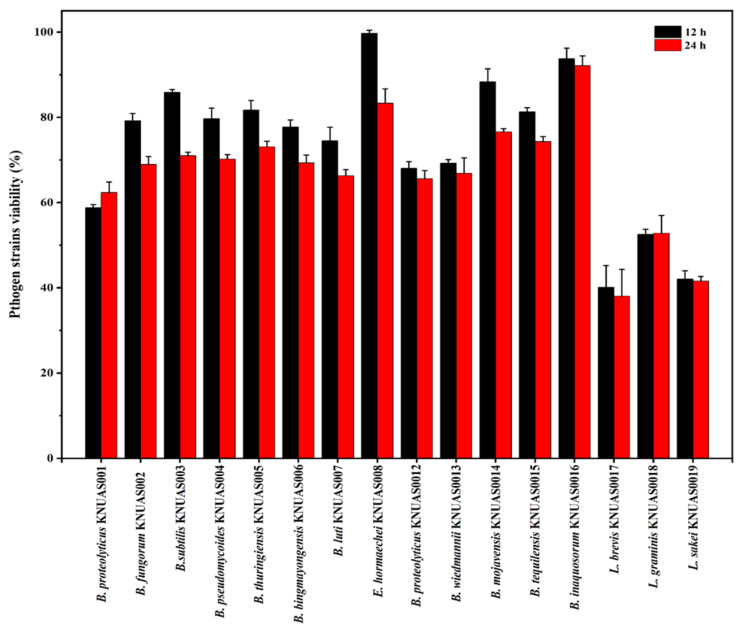
Antibacterial activity of cell free supernatant of isolated bacterial strains against *Escherichia coli* (*E. coli*).

The antibacterial property of CFs of bacterial strains was evaluated with Gram-positive (*S. aureus*) and Gram-negative bacteria (*E. coli*) (Figure 4 and Figure 5). Results found that cell-free supernatant of *Lactobacillus* sp. considerably inhibited pathogenic growth, while cell-free supernatant of *Bacillus* sp. was moderately inhibited when incubated for 24 h. Accordingly, earlier studies reported that *B. subtilis*, *B. mojavensis*, and *B. inaquosorum* showed broad-spectrum antibacterial and antifungal activity [64,65,66]. In addition, earlier studies confirmed that *B. subtilis* and *B. mojavensis* had considerable probiotic potential with antibacterial activity against pathogenic bacteria such as *S. aureus* [41,67]. *B. proteolyticus* and *B. thuringiensis* exhibited pathogenic bacterial inhibition, and the production of antimicrobial peptide bacteriocin from *B. thuringiensis* was reported [68]. Further, the antilisterial peptide (Subtilosin A) showed the inhibition of invasion of the pathogen on human cells, which was isolated from *B. tequilensis* FR9 [69]. However, *E. hormaechei* KNUAS008 showed no significant inhibition of pathogenic growth. The 24 h incubation of CFs of *L. brevis* KNUAS017 considerably inhibited the growth of *S. aureus* and *E. coli* (51.59% and 61.96%), followed by *L. sakei* KNUAS019 (39.54% and 58.42%) and *L. graminis* KNUAS018 (41.56% and 47.19%), respectively. Similarly, the earlier study reported that *L. crispatus* strain exhibited significant inhibitory activity among the other LAB stains when co-cultured with a different bacterial pathogen, which might have a higher content of bacteriocin [70]. Similarly, several studies confirmed the antibacterial efficiency of LAB [58,71]. These results indicated that the antibacterial activity highly depends on the strains and their bioactive metabolites.

## 3. Materials and Methods

### 3.1. Isolation of Probiotic Bacterial Strains and Culture Condition

The bacterial cultures were isolated from the 14 types of fermented samples, including homemade soybean paste, commercial soybean paste, cabbage kimchi, green onion kimchi, leaf mustard kimchi, ginger (*Zingiber officinale*) juice, quince (*Cydonia oblonga*) fruit juice, plum (*Prunus domestica*) fruit juice, crimson glory vine (*Vitis coignetiae*) fruit juice, Korean bramble (*Rubus coreanus*) fruit juice, Japanese apricot (*Prunus mume*) red fruit juice, David’s peach (*Prunus davidiana*) fruit juice, *Prunus mume* green fruit juice (old), and *Prunus mume* green fruit juice (fresh) (Figure S1), were collected from different places in the Republic of Korea. For the isolation, fruit juices (1 mL), soybean paste (1 g), and kimchi (1 g) were inoculated in MRS (de Man, Rogosa, Sharpe) broth purchased from Oxoid LTD, England. The sample inoculated broth was incubated at 37 °C for 24 h under an anaerobic condition. Then, each sample was serially diluted (10^−1^ to 10^−5^) by the standard serial dilution method. At each concentration, 50 µL of the sample was inoculated on an MRS (de Man, Rogosa, Sharpe) agar purchased from Oxoid LTD, England. Then, the plates were incubated at 37 °C for 24–48 h. Further, morphologically distinct colonies were sub-cultured onto MRS agar plates, and pure cultures were stored for further analysis. To determine the lactic acid production, bacterial strains were inoculated in 0.12 g/L of bromocresol purple (Sigma-Aldrich, St. Louis, MO, USA), and MRS agar medium, pH 6.8, was added. The formation of the yellow zone around the colony was a visual indication of lactic acid production.

### 3.2. Identification of Bacterial Strains from Fermented Korean Foods

For identification, a total of 16 pure bacterial isolates were subjected to morphological and 16S rRNA sequencing analysis. Morphological characteristics were determined by Gram staining analysis. In molecular identification, the genomic DNA was extracted from the selected bacterial isolates using a bacterial DNA extraction kit (GenelixTM, Republic of Korea). Further, the isolated bacterial DNA was amplified using standard forward primer 27F (5′-AGA GTT TGA TCM TGG CTC AG-3′) and reverse primer 1492R (5′-TAC GGY TAC CTT GTT ACG ACT T-3′) by polymerase chain reaction (PCR). The PCR condition was used for pre-denaturation at 90 °C for 3 min, denaturation at 95 °C for the 30s, annealing at 56 °C for 30 s, and elongation at 72 °C for 60 s, 30 cycles, with a final extension at 72 °C for 10 min. The obtained PCR product was separated using agarose gel (1.2%) electrophoresis and purified. Then, 16S rDNA sequences were performed at COSMOGENTECH, Republic of Korea. The obtained 16S rDNA sequences were used for the identification of bacterial isolates by nucleotide BLAST search, and then, these sequences were deposited at the NCBI Gene bank (https://www.ncbi.nlm.nih.gov/ accessed on 21 January 2022). The phylogenetic relationship among the sequences was determined using a neighbor-joining method by MEGA X software (version 11).

### 3.3. Resistance to Biological Barriers

#### 3.3.1. Tolerance in Gastric Juice and Intestinal Fluids

Bile salts and pancreatin from the porcine pancreas were purchased from Sigma-Aldrich (St. Louis, MO, USA). Pepsin from pig gastric mucosa was purchased from Roche (Basel, Switzerland). The simulated gastric juice and intestinal fluid tolerance of bacterial strains were determined according to the earlier report [72]. In brief, the bacterial strains were cultured in 30 mL of MRS broth and incubated in an incubator overnight at 37 °C. After incubation, the bacterial cells were collected by centrifugation at 6000× *g* for 10 min, washed with phosphate buffer saline (PBS, pH 7.4), and suspended in 10 mL of PBS. For simulated gastric juice tolerance, 1 mL of bacterial cell suspension was further suspended and incubated for 3 h with 10 mL of PBS (pH 3.0) containing pepsin (0.3%) and NaCl (0.5%). For intestinal fluid tolerance, the bacterial cell suspension was resuspended and incubated for 4 h with 10 mL PBS (pH 8.0) containing pancreatin (0.1%) and bile salts (1%). Bacterial tolerance at each condition was also determined by broth dilution assay by measuring the optical density (OD) at 600 nm under a UV–visible spectrophotometer and inoculating 50 µL of cell suspension onto the MRS agar plate. After incubation at 37 °C, the plates were enumerated, and the results were expressed as log CFU/mL.

#### 3.3.2. Autoaggregation and Coaggregation Ability

The overnight cultured bacterial cells were collected, washed by centrifugation at 6000× *g* for 10 min, and resuspended in PBS (pH 7.4). Autoaggregation was determined by measuring the suspended bacterial cells absorbance (OD at 600 nm) at each predetermined time interval (1, 3, and 6 h), and the plates were kept in an incubator at 37 °C without any disturbance during the experiment. For coaggregation analysis, the bacterial cells were prepared according to the autoaggregation assay. Further, bacterial cells were resuspended in PBS and co-incubated with a cell suspension of *Staphylococcus aureus* (ATCC 19095) under the above-mentioned conditions. The autoaggregation and coaggregation percentages were determined according to the earlier work [61].

#### 3.3.3. Hydrophobicity

To understand the microbial adhesion in the intestine, the bacterial cells were incubated with non-polar solvents and evaluated for their cell surface hydrophobicity. In brief, overnight bacterial cultures were collected by centrifugation at 6000× *g* for 10 min. Then, cells were washed and resuspended with 10 mL of PBS (pH 7.4). Next, 1 mL of bacterial cell suspensions (10^8^ CFU/mL) were mixed with 3 mL of organic solvent (Xylene (98.5%)) and vortex for 60 s. Then, the bacterial and organic solvent mixture was left at room temperature for 60 min. After phase separation, the upper organic phase was removed, and the lower aqueous phase was measured at 600 nm and the hydrophobicity calculated according to an earlier report [73].

### 3.4. Safety Assessment

#### 3.4.1. Hemolytic Property

Hemolytic activity was determined by streaking the bacterial strains on a blood base agar medium supplemented with 5% (*v*/*v*) of sheep blood. All the plates were incubated at 37 °C for 24 h. Hemolytic properties of bacterial strains were determined based on the following changes: α-hemolysis (green color in the medium); γ-hemolysis (no changes), considered non-hemolytic; and β-hemolysis (blood lysis, clear zone), considered hemolytic [74].

#### 3.4.2. Antibiotic Susceptibility

Tetracycline hydrochloride (TCH), erythromycin (ERY), ampicillin sodium salt (AMP), gentamicin sulfate (GEN), and vancomycin hydrochloride (VAN) were purchased from Sigma-Aldrich (St. Louis, MO, USA). The antibiotic sensitivity of bacterial strains was evaluated by the disc diffusion method according to the regulations of the Clinical and Laboratory Standards Institute (CLSI) [75]. The overnight cultured bacterial cell counts were adjusted to 1 × 10^7^ CFU/mL and inoculated on MHA plates. Afterward, antibiotic discs (TCH (30 µg), VAN (30 µg), ERY (15 µg), GEN (10 µg), and AMP (10 µg)) were placed on the bacteria inoculated medium. Then, the plates were incubated at 37 °C for 24 h. The results with a zone of inhibition ≤15 mm were considered to be resistant, 15–21 mm to be intermediate, and ≥21 mm to be susceptible [75]. Experiments were repeated three times.

### 3.5. Characterization of Probiotic Potential

#### 3.5.1. Antioxidant Activity

The antioxidant activity of bacterial strains was determined by DPPH and ABTS radical scavenging assay [32,76]. For 2,2-diphenyl-1-picrylhydrazyl (DPPH) (Sigma-Aldrich, St. Louis, MO, USA) assay, 100 µL of bacterial suspension (1 × 10^9^ CFU/mL) was mixed with DPPH (100 µM) solution, incubated for 15 min in dark condition, the pellet removed by centrifugation, and the supernatant measured at 517 nm by UV–vis spectrophotometer. For 2,2′-Azino-bis(3-ethylbenzothiazoline-6-sulfonic acid) diammonium salt (ABTS) (Sigma-Aldrich, St. Louis, MO, USA) radical scavenging assay, 100 µL of bacterial suspension mixed with 100 µL of ABTS^+^ solution was prepared according to the earlier report [77] and then incubated for 10 min at room temperature in a dark condition. ABTS^+^ scavenging ability was determined by measuring the absorbance of 734 nm. The percentage of DPPH and ABTS^+^ scavenging activity was determined according to an earlier report [76].

#### 3.5.2. Preparation of Culture Free Supernatant

The bacterial strains were cultured in MRS broth at 37 °C for 24 h. The cell-free culture supernatant was collected from bacterial cell suspension cultures by centrifugation at 15,000× *g* for 10 min. To inactivate the organic acid in the culture supernatant, it was aseptically neutralized using NaOH (1 M) at pH 6.5 [78]. Further, the culture supernatant was filtered using a 0.22 µm syringe filter to obtain the cell-free supernatant.

#### 3.5.3. Antibacterial Properties

The antibacterial activity of culture supernatant was determined against Gram-positive (*S. aureus*; ATCC 19095) and Gram-negative (*E. coli*; ATCC 43888) pathogens by agar well diffusion and broth dilution method. For agar well diffusion assay, pathogenic bacteria were initially cultured in nutrient broth (NB) medium overnight at 37 °C. The bacterial cells (1 × 10^9^ CFU/mL) were inoculated onto an MHA plate by sterile cotton swap uniformly, then wells were made using a cork borer. After 50 µL of each bacterial culture supernatant was added to each well, the plates were incubated overnight at 37 °C. Antibacterial ability is represented in terms of the zone of inhibition. For the broth dilution assay, pathogenic bacterial cells (1 × 10^4^ CFU/mL) were inoculated in BHI medium along with culture supernatant (30% *v*/*v*), added to 96-well plates, and kept at 37 °C for 24 h. The antibacterial ability was determined by measuring the absorbance at 620 nm. The percentage of growth inhibition is represented by the percentage of optical density (OD) according to the earlier report [78].

### 3.6. Statistical Analysis

The experimental results were expressed mean ± standard deviation. The statistical significance was determined through a one-way analysis of variance (ANOVA). *p*-value less than 0.05 is considered statistically significant.

## 4. Conclusions

In this study, we isolated sixteen bacterial isolates belonging to three genera, including *Lactobacillus* (3 strains), *Bacillus* (12 strains), and *Enterobacter* (1 strain), from Korean fermented food samples; identified using 16S rRNA gene sequencing; and studied their probiotic potential. Bacterial strains such as *B. subtilis* KNUAS003, *B. proteolyticus* KNUAS012, *L. brevis* KNUAS017, *L. graminis* KNUAS018, and *L. sakei* KNUAS019 exhibited several promising probiotic properties, including tolerance to the gastric environment (simulated gastric juice, bile salts, pancreatin) autoaggregation, coaggregation, hydrophobicity, and non-hemolytic activity. Further, isolated strains showed antibiotic susceptibility and resistance that may be used to develop a controlled therapeutic approach. Additionally, the antibacterial and antioxidant properties of probiotics permitted their utilization and commercialization.

## Figures and Tables

**Table 1:** List of isolated bacterial strains from fermented Korean foods with 16S rRNA species identification and GenBank accession number. According to the new taxonomy of *Lactobacillus*, *Lactobacillu brevis*, *Lacotbacillus graminis*, and *Lactobacillus sakei* named as *Levilactobacillus brevis*, *Latilactobacillus graminis*, and *Latilactobacillus sakei*, respectively.

Strains	Organism	Isolation Source	GenBank Accession Number
KNUAS001	*Bacillus proteolyticus*	Fruit juice of *Prunus domestica*	OM327557
KNUAS002	*Bacillus fungorum*	Rhizome juice of *Zingiber officinale*	OM327558
KNUAS003	*Bacillus subtilis*		OM327559
KNUAS004	*Bacillus pseudomycoides*	Commercial soybean paste	OM327560
KNUAS005	*Bacillus thuringiensis*		OM327561
KNUAS006	*Bacillus bingmayongensis*		OM327562
KNUAS007	*Bacillus luti*		OM327563
KNUAS008	*Enterobacter hormaechei*	Fruit juice of red *Prunus mume*	OM327564
KNUAS012	*Bacillus proteolyticus*	Homemade soybean paste	OM327568
KNUAS013	*Bacillus wiedmannii*		OM327569
KNUAS014	*Bacillus mojavensis*	Green onion Kimchi	OM327570
KNUAS015	*Bacillus tequilensis*		OM327571
KNUAS016	*Bacillus inaquosorum*	Mustard leaf Kimchi	OM327572
KNUAS017	*Lactobacillu brevis* (*Levilactobacillus brevis*)		OM327573
KNUAS018	*Lacotbacillus graminis* (*Latilactobacillus graminis*)	Cabbage Kimchi	OM327574
KNUAS019	*Lactobacillus sakei* (*Latilactobacillus sakei*)		OM327575

**Table 2:** Tolerance of isolated bacterial strains from fermented Korean foods to simulated gastric juice and intestinal fluid. The different superscript letters follow values indicating the significance among the samples (*p* < 0.05).

Bacterial Isolate	Cell Viability (log10 CFU/mL)				
	Simulated Gastric Juice at pH 3.0		Intestinal Fluid (Bile Salts (1%) and Pancreatin (0.1%))		
	1 h	3 h	1 h	2 h	4 h
*B. proteolyticus* KNUAS001	6.72 ± 0.04 ^b^	7.42 ± 0.05 ^b^	7.95 ± 0.02 ^a^	7.54 ± 0.02 ^a^	6.46 ± 0.06 ^a^
*B. fungorum* KNUAS002	6.93 ± 0.07 ^b^	7.89 ± 0.08 ^b^	6.96 ± 0.03 ^b^	6.47 ± 0.04 ^b^	6.19 ± 0.07 ^a^
*B. subtilis* KNUAS003	5.84 ± 0.02 ^c^	5.95 ± 0.02 ^d^	6.73 ± 0.02 ^b^	6.55 ± 0.03 ^b^	6.21 ± 0.05 ^a^
*B. pseudomycoides* KNUAS004	7.26 ± 0.05 ^a^	8.12 ± 0.08 ^a^	7.45 ± 0.04 ^a^	7.24 ± 0.05 ^a^	6.34 ± 0.02 ^a^
*B. thuringiensis* KNUAS005	6.21 ± 0.03 ^b^	6.76 ± 0.03 ^c^	6.21 ± 0.03 ^b^	6.12 ± 0.03 ^b^	6.02 ± 0.07 ^a^
*B. bingmayongensis* KNUAS006	7.21 ± 0.05 ^a^	8.14 ± 0.07 ^a^	7.45 ± 0.05 ^a^	7.08 ± 0.02 ^a^	6.28 ± 0.05 ^a^
*B. luti* KNUAS007	6.58 ± 0.02 ^b^	6.97 ± 0.03 ^c^	7.87 ± 0.02 ^a^	7.52 ± 0.04 ^a^	5.84 ± 0.04 ^b^
*E. hormaechei* KNUAS008	6.78 ± 0.04 ^b^	7.05 ± 0.05 ^b^	6.38 ± 0.04 ^b^	6.21 ± 0.08 ^b^	5.97 ± 0.03 ^b^
*B. proteolyticus* KNUAS012	7.46 ± 0.07 ^a^	6.59 ± 0.04 ^c^	7.46 ± 0.07 ^a^	7.07 ± 0.06 ^a^	6.54 ± 0.07 ^a^
*B. wiedmannii* KNUAS013	7.25 ± 0.08 ^a^	5.37 ± 0.01 ^d^	6.28 ± 0.04 ^b^	6.94 ± 0.02 ^b^	6.24 ± 0.08 ^a^
*B. mojavensis* KNUAS014	5.87 ± 0.04 ^c^	6.64 ± 0.05 ^c^	6.36 ± 0.02 ^b^	6.22 ± 0.07 ^b^	5.65 ± 0.05 ^b^
*B. tequilensis* KNUAS015	6.79 ± 0.02 ^b^	3.47 ± 0.02 ^e^	5.48 ± 0.05 ^c^	5.25 ± 0.05 ^c^	5.15 ± 0.04 ^b^
*B. inaquosorum* KNUAS016	7.86 ± 0.04 ^a^	8.25 ± 0.08 ^a^	7.98 ± 0.03 ^a^	7.74 ± 0.01 ^a^	6.86 ± 0.02 ^a^
*L. brevis* KNUAS017	6.31 ± 0.09 ^b^	6.17 ± 0.04 ^c^	6.24 ± 0.02 ^b^	6.09 ± 0.07 ^b^	5.37 ± 0.01 ^b^
*L. graminis* KNUAS018	2.48 ± 0.04 ^e^	1.56 ± 0.05 ^f^	2.81 ± 0.07 ^e^	2.61 ± 0.08 ^d^	2.21 ± 0.08 ^c^
*L. sakei* KNUAS019	3.48 ± 0.03 ^d^	0.8 ± 0.02 ^g^	3.65 ± 0.04 ^d^	2.82 ± 0.04 ^d^	2.14 ± 0.05 ^c^

**Table 3:** Autoaggregation, coaggregation, and hydrophobicity of isolated bacterial strains from fermented Korean foods to simulated gastric juice and bile salts. The different superscript letters follow values indicating the significance among the samples (*p* < 0.05).

Bacterial Isolates	Autoaggregation (%)			Coaggregation (%)			Hydrophobicity (%)
	1 h	3 h	6 h	1 h	3 h	6 h	Xylene
*B. proteolyticus* KNUAS001	3.87 ± 0.42 ^g^	13.83 ± 0.21 ^e^	34.44 ± 0.59 ^e^	0.62 ± 5.73 ^h^	9.61 ± 0.24 ^f^	30.02 ± 0.33 ^d^	7.44 ± 0.75 ^d^
*B. fungorum* KNUAS002	19.40 ± 0.55 ^b^	30.66 ± 0.25 ^b^	46.30 ± 0.14 ^d^	4.69 ± 0.40 ^f^	19.85 ± 0.08 ^e^	50.67 ± 0.26 ^b^	5.26 ± 1.28 ^e^
*B. subtilis* KNUAS003	18.71 ± 0.38 ^c^	33.67 ± 0.26 ^a^	52.27 ± 0.23 ^c^	36.13 ± 0.46 ^a^	39.75 ± 0.23 ^b^	63.84 ± 0.19 ^a^	1.64 ± 0.38 ^f^
*B. pseudomycoides* KNUAS004	18.60 ± 0.30 ^c^	24.89 ± 0.21 ^c^	49.08 ± 0.16 ^d^	5.82 ± 0.13 ^f^	22.37 ± 0.39 ^d^	46.09 ± 0.26	15.41 ± 0.40 ^b^
*B. thuringiensis* KNUAS005	10.62 ± 0.39 ^d^	15.69 ± 0.41 ^e^	49.32 ± 0.04 ^d^	1.25 ± 0.24 ^g^	24.25 ± 0.22 ^c^	46.44 ± 0.45 ^c^	11.96 ± 0.73 ^c^
*B. bingmayongensis* KNUAS006	6.62 ± 0.08 ^f^	8.85 ± 0.12 ^g^	49.73 ± 0.10 ^d^	1.61 ± 0.44 ^g^	24.34 ± 0.72 ^c^	51.63 ± 0.35 ^b^	0.09 ± 0.83 ^g^
*B. luti* KNUAS007	2.54 ± 0.63 ^h^	20.41 ± 0.34 ^d^	48.86 ± 0.14 ^d^	2.68 ± 0.28 ^g^	20.82 ± 0.06	47.67 ± 0.27 ^c^	13.17 ± 0.76 ^c^
*E. hormaechei* KNUAS008	19.45 ± 0.53 ^b^	26.19 ± 0.38 ^c^	56.85 ± 0.18 ^c^	26.02 ± 0.49 ^b^	48.46 ± 0.33 ^a^	62.58 ± 0.55 ^a^	0.24 ± 0.10 ^g^
*B. proteolyticus* KNUAS012	3.08 ± 0.30 ^g^	10.75 ± 0.31 ^f^	33.73 ± 0.12 ^e^	8.76 ^e^	16.03 ^e^	49.12 ± 0.34 ^c^	12. 46 ± 0.06 ^c^
*B. wiedmannii* KNUAS013	1.52 ± 0.22 ^i^	11.04 ± 0.33 ^f^	51.35 ± 0.27 ^c^	0.39 ± 6.04	9.68 ± 0.29 ^f^	50.39 ± 0.36	4.67 ± 0.17 ^e^
*B. mojavensis* KNUAS014	7.24 ± 0.44 ^e^	8.58 ± 0.27 ^g^	56.98 ± 0.10 ^c^	14.76 ± 0.12 ^d^	20.11 ± 0.40 ^e^	61.67 ± 0.39 ^a^	1.78 ± 1.00 ^f^
*B. tequilensis* KNUAS015	22.63 ± 0.43 ^a^	24.71 ± 0.18 ^c^	64.56 ± 0.14 ^a^	21.21 ± 0.29 ^c^	34.15 ± 0.55 ^b^	63.64 ± 0.35 ^a^	7.29 ± 0.63 ^d^
*B. inaquosorum* KNUAS016	8.51 ± 0.20 ^e^	10.82 ± 0.18 ^f^	61.63 ± 0.19 ^b^	5.83 ± 0.19 ^f^	7.36 ± 0.42 ^f^	57.63 ± 0.48 ^b^	16.76 ± 0.16 ^b^
*L. brevis* KNUAS017	8.58 ± 0.18 ^e^	21.58 ± 0.18 ^d^	29.98 ± 0.09 ^e^	0.75 ± 0.21 ^h^	27.45 ± 0.26 ^c^	33.86 ± 0.12 ^d^	25.64 ± 0.28 ^a^
*L. graminis* KNUAS018	0.95 ± 0.17 ^i^	3.06 ± 0.33 ^h^	31.11 ± 0.19 ^e^	5.56 ^f^	12.14 ± 0.29 ^f^	45.71 ± 0.23 ^c^	18.81 ± 1.36 ^b^
*L. sakei* KNUAS019	7.01 ± 0.23 ^e^	26.06 ± 0.11 ^c^	66.55 ± 0.33	0.66 ± 0.29 ^h^	24.71 ± 0.21 ^c^	55.69 ± 0.30 ^b^	14.91 ± 0.48 ^c^

**Table 4:** Antibiotic susceptibility of isolated bacterial strains. Tetracycline hydrochloride (TCH, 30 µg); vancomycin hydrochloride (VAN, 30 µg); erythromycin (ERY, 15 µg); gentamicin sulfate (GEN, 10 µg); ampicillin sodium salt (AMP, 10 µg). ≤15 mm, resistance; 15–21 mm, intermediate; and ≥21 mm, susceptible. The different superscript letters follow values indicating the significance among the samples (*p* < 0.05).

	Zone of Inhibition (mm)				
Bacterial Isolates	TCH	VAN	ERY	GEN	AMP
*B. proteolyticus* KNUAS001	22.2 ± 0.4 ^b^	10.2 ± 0.3 ^c^	23.2 ± 0.3 ^c^	14.1 ± 0.1 ^b^	9.2 ± 0.3 ^d^
*B. fungorum* KNUAS002	23.7 ± 0.4 ^b^	14.9 ± 0.1 ^b^	22.9 ± 0.1 ^c^	14.3 ± 0.4 ^b^	8.2 ± 0.4 ^d^
*B. subtilis* KNUAS003	18.7 ± 1.1 ^c^	11.3 ± 0.4 ^b^	19.3 ± 0.5 ^d^	13.3 ± 0.5 ^b^	12.1 ± 0.1 ^d^
*B. pseudomycoides* KNUAS004	25.9 ± 0.1 ^b^	17.7 ± 0.4 ^a^	25.8 ± 0.3 ^a^	18.2 ± 0.3 ^a^	39.7 ± 0.4 ^a^
*B. thuringiensis* KNUAS005	22.2 ± 0.4 ^b^	17.8 ± 0.3 ^a^	27.1 ± 0.1 ^a^	12.2 ± 0.3 ^c^	21.8 ± 1.1 ^b^
*B. bingmayongensis* KNUAS006	12.2 ± 0.3 ^d^	13.1 ± 0.1 ^b^	28.1 ± 0.1 ^a^	21.5 ± 0.7 ^a^	10.1 ± 0.2 ^d^
*B. luti* KNUAS007	31.7 ± 0.4 ^a^	19.1 ± 0.2 ^a^	10.1 ± 0.1 ^e^	17.2 ± 0.3 ^a^	20.9 ± 0.1 ^b^
*E. hormaechei* KNUAS008	18.3 ± 0.4 ^c^	12.2 ± 0.4 ^c^	22.2 ± 0.3 ^c^	12.3 ± 0.5 ^c^	22.1 ± 0.1 ^b^
*B. proteolyticus* KNUAS012	28.8 ± 0.2 ^a^	0	22.3 ± 0.4 ^c^	17.4 ± 0.6 ^a^	39.4 ± 0.9 ^a^
*B. wiedmannii* KNUAS013	25.3 ± 0.4 ^b^	18.2 ± 0.4 ^a^	23.1 ± 0.1 ^c^	0	41.3 ± 0.9 ^a^
*B. mojavensis* KNUAS014	0	0	0	0	0
*B. tequilensis* KNUAS015	10.9 ± 0.1 ^d^	14.1 ± 0.2 ^b^	16.2 ± 0.3 ^d^	12.1 ± 0.1 ^c^	18.1 ± 0.1 ^c^
*B. inaquosorum* KNUAS016	0	0	0	0	0
*L. brevis* KNUAS017	0	0	0	0	0
*L. graminis* KNUAS018	24.8 ± 0.2 ^b^	0	24.9 ± 0.1 ^c^	0	23.2 ± 0.3 ^b^
*L. sakei* KNUAS019	21.4 ± 0.6 ^b^	0	17.1 ± 0.2 ^d^	0	18.0 ± 0.0 ^c^

## Data Availability

The data presented in this study are available in supplementary material.

## Supplementary Materials

The supporting information can be downloaded at: https://www.mdpi.com/article/10.3390/ijms231710062/s1.
