# Supplementary material for: Antioxidant and Antibacterial Effects of Potential Probiotics Isolated from Korean Fermented Foods
Source: Int J Mol Sci. 2022 Sep 2;23(17):10062. doi: 10.3390/ijms231710062 (PMC9455991; doi:10.3390/ijms231710062)
Supplement: Supplementary file 1 [file ijms-23-10062-s001.zip › ijms-1861115-supplementary.pdf]

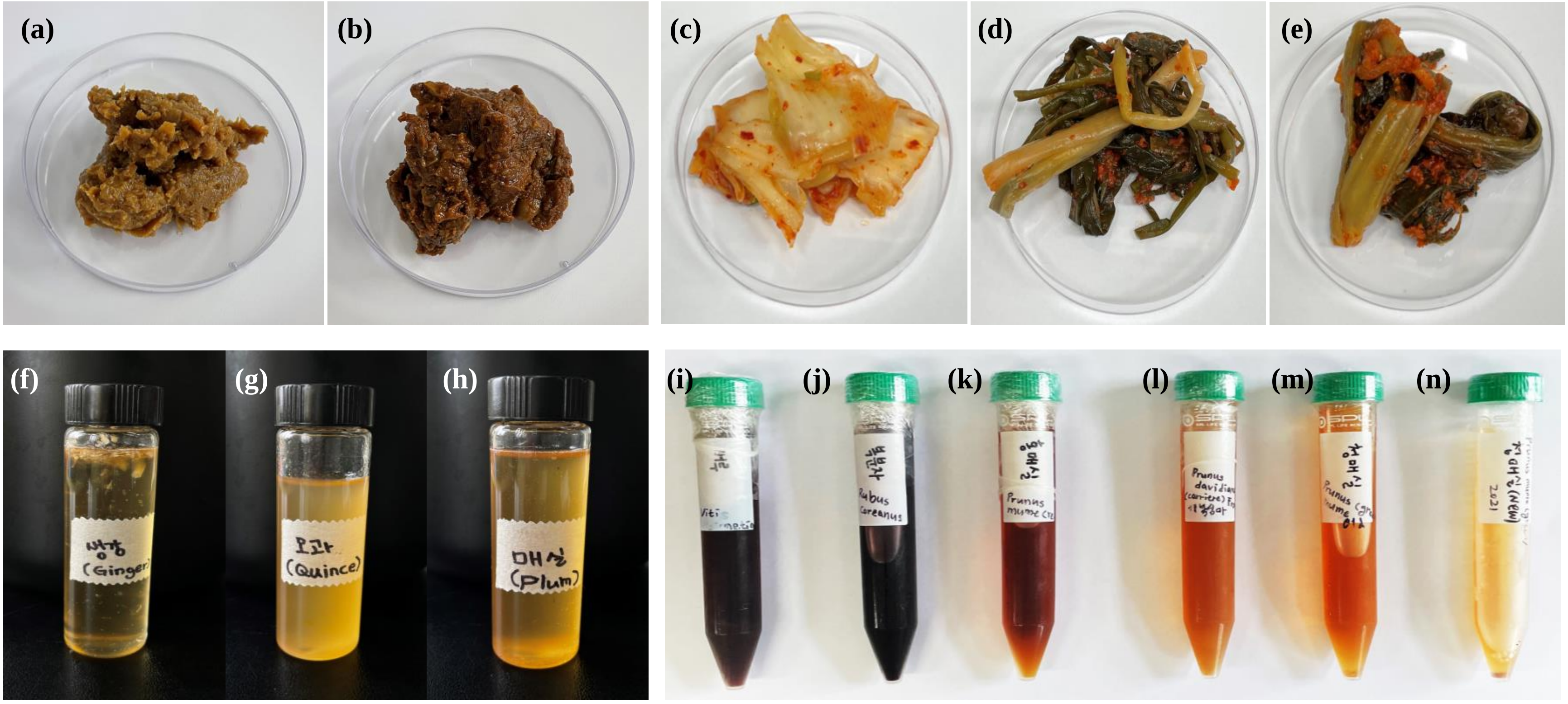

Figure S1. Fermented Korean food samples used for probiotics isolation. Home made soybean paste (HMS) – a; commercial Soybean paste (CS) – b; cabbage kimchi (CAK) – c; green onion kimchi (GOK) – d; leaf mustard kimchi (LMK) – e; ginger (*Zingiber officinale*) juice – f; quince (*Cydonia oblonga*) fruit juice – g; plum (*Prunus domestica*) fruit juice – h; crimson glory vine (*Vitis coignetiae*) fruit juice – *i;* Korean bramble (*Rubus coreanus*) fruit juice – *j;* Japanese apricot (*Prunus mume*) red fruit juice – *k;* David's peach (*Prunus davidiana*) fruit juice – *l; Prunus mume* green fruit juice (old) – *m; Prunus mume* green fruit juice (fresh) – *n.*

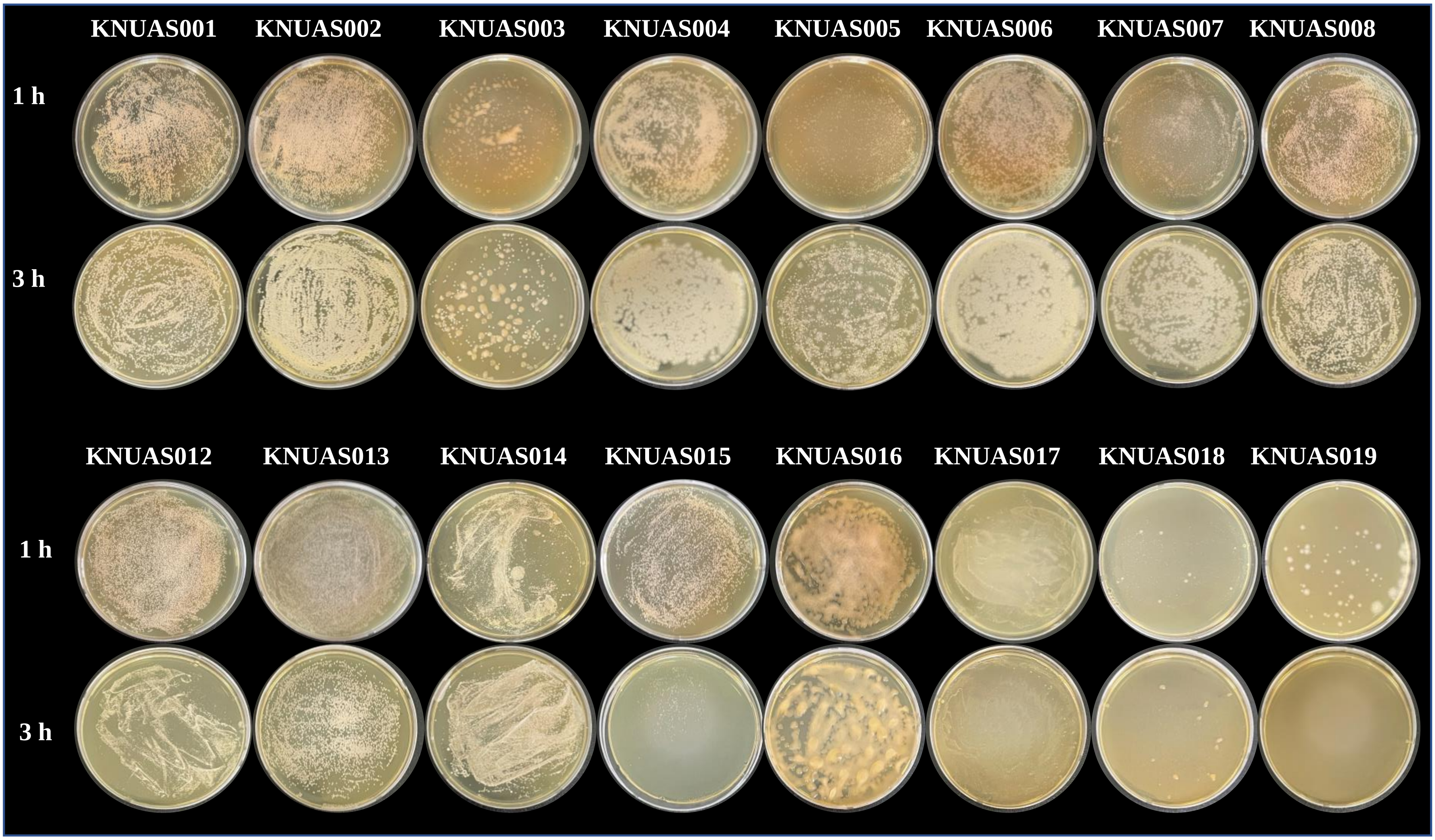

Figure S2. Simulated gastric juice environment tolerance of isolated bacterial strains from fermented Korean foods. Cell viability of bacterial strains on simulated gastric juice environment at 1 h and 3 h.

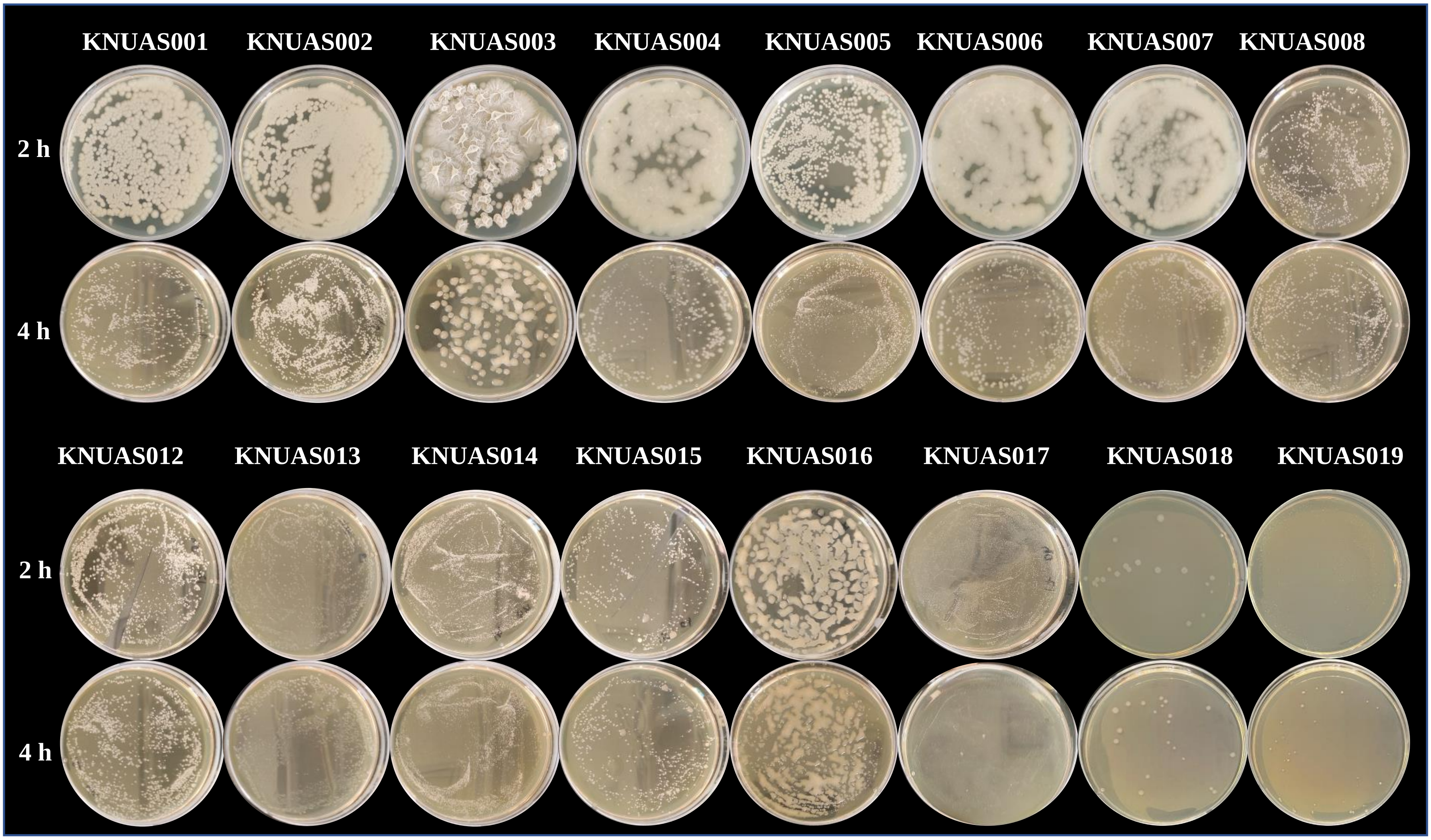

Figure S3. Bile salts environment tolerance of isolated bacterial strains from fermented Korean foods. Cell viability of bacterial strains on simulated gastric juice environment at 2 h and 4 h.

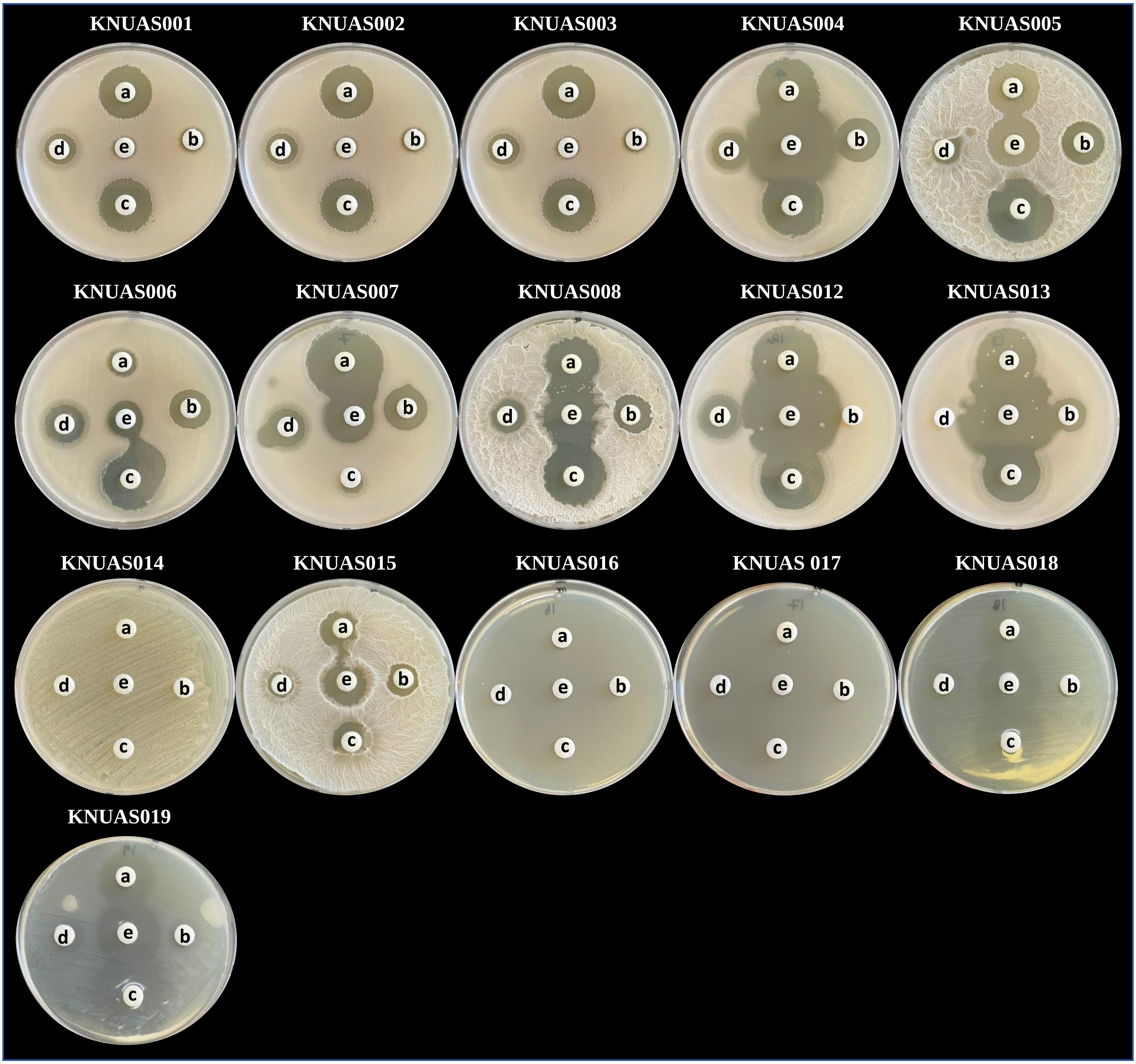

Figure S4. Antibiotic susceptibility of isolated bacterial strains. Tetracycline hydrochloride (TCH, 30 µg) – a; vancomycin hydrochloride (VAN, 30 µg) – b; erythromycin (ERY, 15 µg) – c; gentamicin sulfate (GEN, 10 µg) – d; ampicillin sodium salt (AMP, 10 µg) – e.
